# A review of frailty instruments in human medicine and proposal of a frailty instrument for dogs

**DOI:** 10.3389/fvets.2023.1139308

**Published:** 2023-06-27

**Authors:** Rachel L. Melvin, Audrey Ruple, Elizabeth B. Pearson, Natasha J. Olby, Annette L. Fitzpatrick, Kate E. Creevy

**Affiliations:** ^1^Department of Small Animal Clinical Sciences, Texas A&M University School of Veterinary Medicine & Biomedical Sciences, College Station, TX, United States; ^2^Department of Population Health Sciences, Virginia-Maryland College of Veterinary Medicine, Virginia Tech, Blacksburg, VA, United States; ^3^Department of Clinical Sciences, NC State University College of Veterinary Medicine, Raleigh, NC, United States; ^4^Department of Family Medicine, University of Washington, Seattle, WA, United States

**Keywords:** aging, age-related disease, geriatric, phenotype, resilience

## Abstract

Over the last few decades, frailty has become a pillar of research and clinical assessment in human gerontology. This complex syndrome, characterized by loss of physiologic reserves leading to decreased resilience to stressors, is of critical importance because it predicts higher risks of poor health outcomes, including mortality. Thus, identifying frailty among the elderly human population has become a key focus of gerontology. This narrative review presents current scientific literature on frailty in both humans and animals. The authors discuss the need for an accessible frailty instrument for companion dogs suitable for general use in veterinary medicine and the advances that would be facilitated by this instrument. A phenotypic frailty instrument for companion dogs, utilizing components that are easily collected by owners, or in the general practice setting, is proposed. The authors elaborate on the domains (physical condition, physical activity, mobility, strength, cognitive task performance, and social behavior), factors that will be included, and the data from the Dog Aging Project that inform each domain.

## 1. Introduction

### 1.1. Definition of frailty

Age carries a widely known association with increased risk for disease and adverse health outcomes. However, chronological age alone is neither a sensitive nor specific predictor of morbidity (1) when assessing health at the level of the individual. In lieu of chronological age, the phenomenon of frailty is now recognized in human gerontology as a superior way to assess the manifestations of aging and associated risks of disease and death within individuals and communities (1–5). Recognition of frailty adds a valuable dimension to the assessment of health and prognosis by documenting states of decline or loss of function which can emerge without any corresponding diagnosis of disease (6, 7). While frailty is associated with age, that association is complex in that individuals manifest frailty to varying degrees and at varying ages (8–11). Furthermore, frailty, unlike chronological age, can sometimes be reversed (12–14).

Frailty, an evolving concept in human gerontology, is a complex, multidimensional syndrome characterized by a loss of resilience to stressors associated with aging (2, 5, 15, 16). Resilience itself is also a multifaceted characteristic which is affected by patient-specific and environmental factors, and which confers both physiological and emotional reserves (4, 17). The underlying etiology of frailty is thought to be the dysregulation of multiple physiologic systems resulting in loss of these reserves (2, 3, 18). Underlying mechanisms of frailty are not yet fully elucidated, and possibilities include sarcopenia (19, 20), low-grade inflammation (2), immune dysfunction (20), endocrinopathies (20, 21), and genetic risk factors (2). Increased resilience has been shown to predict recovery from illness and/or injury and improved quality of life in individuals living with chronic conditions (17). By contrast, frailty is associated with an increased risk of health problems including falls/fractures (15, 20, 22), hospitalization (15, 22, 23), functional decline/disability (15, 20, 22), the requirement for long-term care or institutionalization (3, 24, 25), and death (15, 16, 22, 24). Because frailty leads to adverse health outcomes, including mortality, identifying frailty among elderly human individuals and populations has become an important focus of gerontology.

### 1.2. Human frailty applications

In 2001, Fried et al. proposed a phenotype of frailty to be applied in community-dwelling populations to identify at-risk individuals. It included components of unintentional weight loss, weakness, exhaustion, slowness, and low physical activity (15). Frailty, defined as the presence of three or more of these five characteristics, was predictive of adverse outcomes in relatively functional geriatric adults (15). Individuals identified as frail were six times more likely to die in the subsequent 3 years compared to their non-frail counterparts (15). The field of frailty has expanded dramatically over the last 20 years, with a multitude of instruments currently available to measure frailty in humans. These instruments typically can be categorized as Frailty Phenotype (FP) or Frailty Index (FI) instruments (25–27) (Table 1). Broadly, the FP model is based on the presence or absence of components that can be physically evaluated, while the FI model includes medical and laboratory findings and assigns numeric scores to those components. FP and FI instruments have been found to be comparable in the prediction of mortality (25, 27) and can be considered complementary in certain situations (26).

**Table 1:** Frailty phenotype (FP) and frailty index (FI) comparison.

Frailty phenotype (FP)	Frailty index (FI)
Presence or absence of specific components, most commonly:	Cumulative score of health deficits, detected by:
Weakness (often based on grip strength)	Overall disease count,
Low mobility (often based on slow walking speed)	Tally of specific diseases,
Low physical activity (kilocalorie expenditure per week)	and/or
Unintentional weight loss	Laboratory abnormalities
Fatigue or exhaustion (self-reported)	
Score is a sum of binary classifications	Score is either continuous, or a ratio of deficits detected within components assessed
Considers frailty to be a decline in physical function	Considers frailty to be an accumulation of health deficits within multiple domains

Frailty models have evolved over the years from physical-only models to ones that include frailty factors from psychological (28, 29), social (1, 29, 30), and environmental domains (31, 32). Currently, there are a plethora of different instruments to detect and measure frailty. A 2019 systematic review of studies evaluating frailty instruments used in human populations found a total of 51 diverse instruments, including numerous instruments using the FP model and many using the FI model (33). These instruments varied greatly in length/duration, use, interpretive categories, information collected, collection method, need for additional diagnostics, areas of investigation, etc. (Table 2). This diversity of frailty instruments is the product of different needs depending on the setting, administrator, available time, and aim of the measurements (42). However, these different instruments classify different subsets of the population as frail (43) which means that investigators must carefully select instruments for target applications. Across studies, the highest prevalence of frailty in human populations is observed in studies using multidimensional instruments (44), suggesting they may be more sensitive. There is also evidence that an increased number of included variables tends to result in a higher agreement between instruments and narrow prediction intervals, and that multidimensional instruments result in less error at the median point of frailty (43).

**Table 2:** Design and intended uses of a sample of frailty instruments in human gerontology.

Number of interpretive categories within the instrument	
	Frail vs. non-frail (34, 35)
	≥3 Levels of frailty (15, 23)
Scale type	
	Dichotomous scale (36–38)
	Ordinal scale (1, 15, 39)
	Continuous scale (28, 29)
Domains included, with most commonly used domains listed first:	
	Physical (15, 23, 37, 39)
	Psychological (28, 29)
	Social (1, 28, 30)
	Environmental (31, 32)
Settings in which instrument is used	
	Community (1, 15, 28, 39)
	Primary care/geriatric practice (29)
	Emergency departments (40)
	General hospitals (41)
	Long-term care facilities/nursing homes (30, 36, 41)

Along with the number of instruments and the domains they cover, the possible settings for the use of frailty assessment are also an expanding area of investigation. The identification of frail individuals informs prognosis and medical options and allows for several types of intervention directed at overall health and frailty itself. In human medicine, frailty can be used to stratify risk profiles and inform decision-making (16, 45). In a study of geriatric people who were treated for pelvic fractures, those identified as frail were at greater risk of mortality at the time of discharge and one-year post-discharge, and were also at risk of having reduced functionality and needing greater assistance at discharge (46). Frailty was associated with an increased risk of serious complications in geriatric trauma patients (47), and frailty status increased the risk of long-term mortality in COPD patients by 80% (48). This prognostic information is valuable for both patients and their families to understand prior to surgery or other medical treatment.

The identification of frail patients is also necessary to take any protective or preventive action, such as adjustments in the typical geriatric medical or surgical protocols to improve outcomes for a specific frail individual, or enrollment in programs targeted at altering the frailty state itself. In human geriatric patients, screening instruments can be utilized to identify frail patients simply to inform their healthcare team so that they can make treatment alterations as needed. One study looking at postoperative survival for human patients undergoing major elective surgical procedures found that simply implementing a frailty screening initiative resulted in improved survival (49). When a patient was identified as frail, anesthesia, surgery, critical care, and palliative care clinicians were informed and, if needed, a modified perioperative plan was developed (49). Decreased mortality using this approach was attributed to multiple factors, including increased vigilance for complications leading to earlier treatment and improved family involvement, leading to improved post-discharge care and social support (49).

As frailty is a dynamic process, influenced by both individual and environmental factors, it is susceptible to intervention. Frailty studies in human medicine have shown that early identification and targeted intervention can delay (50, 51), prevent (52), or reverse the progression of frailty (12–14). Early diagnosis is often noted to be important for a more positive outcome (12, 14, 51). Interventions are wide-ranging, including nutritional supplementation, medications, and exercise programs, among others (2). In a study of frailty scores, life-space (a measure of the geographical space in which a person’s life takes place) and quality of life, Chitalu and colleagues found that among people with high frailty scores, those with high life-space had better quality of life than similarly frail people with lower life-space; by contrast, life-space was a less relevant factor to quality of life among non-frail individuals (53). This finding suggests that specific interventions such as increasing life-space, may preferentially benefit frail individuals compared to their potential benefit on the greater geriatric population overall. An exploratory analysis of the MoveStrong exercises suggested improvement in frailty indicators (gait speed, balance, sit-to-stand functioning) and health-related quality of life in both frail and pre-frail individuals who used these exercises (54).

It is clear that instruments deployed in the assessment of human frailty fulfill a variety of roles. Some are used for screening large community populations for both pre-frailty and frailty (15), some are used within long-term care facilities (30, 36, 41) and others are used in healthcare facilities (29, 40, 41) and may include components of diagnostic data (34, 55, 56). The diverse instruments range from simple to complex and include assessment of numerous and varying domains. There are challenges inherent to the design of some instruments, and the mere fact that so many instruments exist creates challenges. The ability of a clinician or investigator to select the ideal human frailty instrument for the setting and goals is valuable, however, the existence of so many instruments and lack of consistency in use precludes large-scale assessment of their performance or comparison between groups of individuals or intervention techniques.

### 1.3. Frailty in other species

The importance and relevance of defining positive and negative trajectories of aging have been recognized widely. Outside of human gerontology, frailty scales developed in the laboratory setting for mice and rats have been shown to predict adverse outcomes (57–60), confirming that frailty is relevant and applicable to other species. By contrast, companion animal assessments rely heavily on owner-reported information. In this vein, veterinary specialties have developed quality of life (QoL) tools that are specific to a condition or body system [e.g., Canine Owner Reported QoL Questionnaire (61), Canine Symptom Assessment Scale (62), and questionnaires for QoL in patients with spinal cord injuries (63), patients with pain secondary to neoplasia (64), and patients undergoing chemotherapy (65)]. QoL questionnaires frequently capture a range of domains that are closely aligned with frailty and demonstrate the feasibility of companion animal frailty assessment by owner-reported metrics. Taking it a step further, McKenzie et al. proposed a Canine Geriatric Syndrome as a framework to evaluate physical, functional, behavioral, and metabolic changes in aging dogs to better understand and investigate the biological aging process; frailty is one of the proposed components of the syndrome (66). Despite this, the study of frailty in veterinary medicine, and specifically in dogs, is still in its infancy.

Hua et al. developed a FP instrument, evaluating five phenotype components derived from those used in human gerontology (67). They extrapolated findings relevant to these five components from the records of physical examinations performed on geriatric service dogs, and found that the presence of more than two of these components was significantly associated with decreased time until death, independent of age, health status, or subclinical and clinical diseases (67). Banzato et al. developed a questionnaire-based FI instrument including 33 health deficits, meant to reflect data collected in a routine health exam (45). Banzato et al.’s FI was shown to have moderate accuracy in predicting short-term mortality (45). In both Hua et al. and Banzato et al.’s work, the prevalence of frailty in dogs was noted to be similar to that of humans (15, 43–45, 67), and the overall mean FI calculated by Banzato et al. was similar to the overall FI for community-dwelling individuals (45, 57). In human gerontology, it has been reported that a FI of 0.7 is considered to be the “threshold when the accumulation of deficits becomes incompatible with life” (45, 68); Banzato et al. noted a similar cut-off point of 0.7 in their FI for dogs (45). Additionally, a new frailty phenotype based on owner responses to simple questions has shown strong predictive power for short term (6 months) mortality in a cohort of healthy senior dogs (69). Collectively, these studies demonstrate that a frailty syndrome similar to that described in human gerontology is also present, and can be assessed, in dogs. A standardized frailty assessment could become as important a tool in geriatric canine health as it is in human healthcare.

## 2. The need for frailty assessment in veterinary medicine

The importance of frailty assessment in human geriatric medicine has expanded as the elderly proportion of the human population has grown (22, 70, 71). Similarly, as the geriatric veterinary patient population expands, due to progress in diagnostic capability and therapeutic options that improve the overall management of pet health (72), the need for a clinically applicable frailty score in veterinary medicine is becoming clear. Age is the greatest risk factor for the development of frailty and, perhaps because of this strong association, the development of frailty and other age-related diseases within an aging individual is often viewed as inevitable (66). The belief that age alone is the cause of poor health outcomes may lead owners to forego opportunities to investigate actual underlying causes and provide potential interventions. This concept of a ‘normal’ state of poor health inevitably associated with aging is a barrier to the progress of geriatric veterinary medicine. The development and wide use of validated, objective frailty instruments in veterinary medicine are needed to advance the study of companion animal gerontology. Specifically, care of the geriatric companion dog is becoming a more important facet of veterinary general and specialty practice (73–75).

The ability to stratify risk profiles within the geriatric companion dog population, as was described above for human gerontology, could significantly impact prognostication and medical care for dogs. Humane euthanasia of companion dogs is a common manner of death, elected by owners due to a variety of reasons including old age, devastating illness or injury, the perception that recovery is unlikely, lack of access to or affordability of needed care, and poor quality of life, among others (76–78). Thus, veterinary medicine is uniquely impacted by the need to provide accurate prognostic information and to combat assumptions of poor prognosis based on age alone, because perceived poor prognosis can lead to elective euthanasia. Owners’ assessments of their dogs’ health-related quality of life (HRQL) have been shown to be heavily influenced by age itself, rather than survivability (79), and perception of poor HRQL could also promote the owner’s choice to euthanize (17, 18, 42, 80). By contrast, the deployment of a validated companion dog frailty instrument would enable studies to determine the actual stratified assessment of the risk of negative outcomes for geriatric dogs as a function of their frailty. Such a tool could then better support owner decision-making, avoiding the assumption of a poor prognosis simply based on the age of the dog. For example, a retrospective study of the outcomes of over 6,000 dogs entered into the Veterinary Committee on Trauma registry found that among moderately injured dogs, geriatric dogs had a significantly higher risk of death despite intervention, or of experiencing euthanasia due to grave prognosis, than their nongeriatric counterparts (81). The higher rate of deaths despite intervention among geriatric dogs suggests that they may have been frailer; by contrast, the higher rate of euthanasia among these dogs suggests that their owners or attending clinicians may have believed their prognosis was worse than the prognosis of younger dogs with similar injuries.

A frailty screening tool for veterinarians to use to easily identify frail dogs would also allow for adjustments in management protocols, from anesthesia to treatment, similar to the previously mentioned study in human geriatric elective surgical patients (49) – and ideally in the future allow veterinarians to more accurately predict recovery from specific events or illnesses. The first step in the ability to make frailty-specific protocol adjustments to improve frail patients’ outcomes is the ability to identify those patients. The identification of frail dogs would also allow for direct intervention in the frailty state as is seen in human medicine (2, 13, 14, 51, 52, 54). For instance, programs to target strength, balance, or maintenance of muscle mass could be implemented in veterinary medicine to directly improve the lives of geriatric companion dogs. Furthermore, there is a growing body of research into strategies to extend lifespan and healthspan in companion dogs (82–85). Lifespan is a challenging target to deploy in such clinical trials because it can be modified by owner-elected euthanasia in response to owner perception of the dog’s status, access to care, and other factors. Widespread adoption of a validated frailty instrument would enable frailty – physiologically meaningful, aging-associated phenotype – to replace lifespan as a valuable endpoint for such clinical trials. The ability to evaluate the effect of individual frailty on outcomes in specific situations and disease conditions as well as to appraise the utility and impact of an interventional program hinges on the ability to recognize and measure frailty. A widely used frailty assessment for dogs would also allow for comparison between medical institutions and different studies, longitudinal monitoring for individual patients, and an endpoint in clinical trials that assess interventions into aging and age-related decline.

## 3. Dog Aging Project proposed frailty instrument for dogs

The ideal companion dog frailty instrument would maximize the benefits while minimizing or eliminating the weaknesses associated with the diverse frailty instruments used in human gerontology (Table 3). As described above, diverse instruments available in human gerontology may satisfy the needs of different users but also make the collection of comparable data among sites or populations challenging. If frailty is to become a common component of the assessment of companion dogs, it would be beneficial to avoid this fragmentation of data by ensuring that a single tool is widely accessible and used in all dimensions of veterinary practice. The Dog Aging Project (DAP) is an open-data, long-term, longitudinal study of the genetic and environmental determinants of healthy aging in companion dogs (100, 101) and the ability to assess frailty in this population is paramount to fulfill the goals of the study. Enrolled dogs represent diverse household environments in all 50 US States and receive medical management from their primary care veterinarians throughout the study. A companion dog frailty instrument that can be used in all the practices in which these dogs receive routine medical attention will create an opportunity for the collection and clinical application of comparable frailty information not just from DAP-enrolled dogs, but from all companion dogs. The DAP has been previously described (100). Briefly, the DAP collects comprehensive information about dietary and exercise management, home environments, and health histories of enrolled dogs and it is likely that some of these experiences could influence, or be influenced by, frailty. The utility of a proposed frailty instrument would be assessed among these diverse groups of dogs, to better describe the manner in which varying attributes and experiences impact the trajectory of frailty in companion dogs.

**Table 3:** Maximizing strengths and avoiding weaknesses of human gerontology frailty assessment in a proposed companion dog frailty instrument.

Strengths	
Human frailty assessment	Proposal
Several key phenotypic factors (unintentional weight loss, self-reported exhaustion, weakness, slow walking speed, poor endurance, and low physical activity) are included in the most commonly used instruments (15, 39, 41, 56, 86–91).	Adapt these key dimensions to dogs
Instruments that include multiple physiologic domains such as illness or comorbidities, functional status for daily activities, use of medications, decreased appetite, nutrition, balance, flexibility, falls, mobility disability, and vision loss (1, 23, 29, 30, 34, 37, 40, 41, 55, 87, 89, 90, 92–94).	Include assessments of physiologic domains relevant to dogs to broaden the assessment
Instruments that include multiple functional (non-medical) domains such as activities of daily living (ADL)/instrumental activities of daily living (IADL), communication (speech/hearing), mood alteration, low motivation, reclusion, cognition, and social support (1, 23, 28–31, 34, 41, 55, 92)	Include assessments of functional domains relevant to dogs to broaden the assessment
Multidimensional instruments (31, 36, 95).	Ensure multi-dimensional components are included to maximize predictive value
Short instruments (15, 39, 87).	Minimize the number of components to decrease the burden on users
Instruments utilizing self-reporting (29, 89, 92).	Select components apparent to dog owners at home
Assessment that can be performed at home, by phone or mail (35, 91–93).	Select components readily assessed by dog owners at home
Instruments that can be performed by individuals without medical training (31, 32, 88).	Select components that can be performed by owners
Instruments that can be performed by primary care providers (29).	Select components that can quickly be performed by veterinary general practitioner
Weaknesses	
Human frailty assessment	Proposal
Lengthy/time-consuming instruments (28, 96, 97).	Ensure that owner or primary care veterinarian can complete in less than 30 min to minimize burden on users
Instruments that include complex measurements including the Progressive Romberg Test, Brachial Ankle Index, and tests using wireless motion sensors (56, 98).	Limit to simple, low-tech measurements to facilitate wide use
Instruments that include medical or laboratory data (albumin, blood pressure – systolic or arterial, red blood cells/hemoglobin counts, etc.) which may be unavailable or expensive to acquire (23, 28, 34, 37, 55, 56, 99).	Limit to externally apparent phenotypic data to avoid invasiveness or added cost
Instruments that require specifically trained personnel to administer (17).	Ensure that minimally trained owners or veterinary personnel can obtain each measurement to facilitate wide use

As discussed above, frailty instruments in human gerontology may be optimized for different settings, and ultimately a three-tiered structure (Screening, Assessment, Triage) for frailty evaluation in dogs seems valuable. The Screening step would enable owners to identify dogs likely to benefit most from frailty assessment, whereas the Triage step could be deployed in urgent medical settings to enable appropriate frailty-based protocol modifications for dogs who had not been previously assessed. Here we propose the design and rationale for an Assessment instrument, the Frailty Instrument for Dogs (FIDo).

Development of the FIDo is guided by several overarching goals, derived from observations about the strengths and weaknesses of various human frailty instruments (Table 3). To ensure that this instrument will be accessible to most veterinarians and owners, it must be easy to use, low-tech, low-cost, and of reasonable length (ideally less than 15 min for completion). To capture the complexity and multifactorial essence of frailty, the instrument must be a multi-dimensional tool, including physical, social, and psychological components. As previously discussed, there are two major models for frailty instruments utilized in human gerontology (FP and FI), however, there is no consensus on the better model. Both have been found to provide comparable predictions of mortality (27), and they may be complementary (26) in providing a more robust understanding of an individual. To achieve the goal that the FIDo is low-tech, low-cost, easily implemented, and widely available and utilized for the assessment of frailty, the DAP elected to build a phenotypic frailty model that includes externally apparent components. Use of a phenotypic instrument will allow the identification of frail patients using information that can be readily collected by owners and veterinarians without the need for medical or diagnostic interactions, that may hinder implementation due to financial or other barriers to care. A phenotypic approach also facilitates the identification of frail individuals that may be free of diagnosed disease (9, 26). Thus, in contrast to Banzato et al.’s FI instrument (45) diagnostic data and multimorbidity will be components of a separate assessment tool in the DAP framework. This multimorbidity tool would be available to use as a complementary assessment tool with FIDo for geriatric care in the future. In contrast to the FP instrument previously reported by Hua et al. (67), the components of the DAP FIDo will be prospectively collected by both owners and veterinarians.

Domains for the DAP FIDo were carefully chosen to reflect those most often used in human gerontology which can be assessed in dogs. Multiple factors within each domain will be included in the initial analysis to determine which are the most relevant phenotypic markers of frailty. The domains that will be included in the DAP FIDo are physical condition, physical activity, mobility, strength, cognitive task performance, and mood and social relations (Table 4). In previous studies of congestive heart failure, diabetes mellitus, and osteoarthritis it was demonstrated that owners are sensitive and accurate reporters of their dog’s health status (102–104), thus owners will serve as primary data sources for most domains. Specific owner-provided information will be extracted from the DAP’s Health and Life Experience Survey (HLES), Annual Follow-Up Survey (AFUS), Canine Social and Learned Behavior Survey (CSLB) (105), Measurement and Mobility Activities (M&M), and Cognitive Tasks (100). Certain factors will also be extracted from veterinary electronic medical records (VEMRs) uploaded by participating owners. These components will initially be evaluated separately to determine which are predictive of mortality in dogs. As each component will be collected longitudinally, we will also assess whether the rate of change of each component is more strongly predictive of mortality than a discrete value at a single time point. All predictive components will be included in the first comprehensive model. Components that are not found to have a relationship with frailty will be removed. Stepwise analysis will allow removal of components that provide redundant information; remaining components will be weighted by a coefficient proportional to the association of each parameter with mortality.

**Table 4:** Dog Aging Project (DAP) proposed companion dog frailty instrument.

Domain	Factor	Source of data	Nature of data
Physical condition	Body condition score (BCS)	VEMR	Ordinal numeric value extracted from records
	Thigh girth deterioration	M&M	Continuous numeric value (centimeters) obtained by owners
	Unintentional weight loss Veterinarian-reported Owner-reported	HLES VEMR	Continuous numeric value (kilograms) extracted from records and reported by owners
Physical activity	Customary activity	HLES AFUS	Ordinal qualitative description reported by owners
Mobility	Gait speed On-leash Off-leash	M&M	Continuous numeric value (seconds) obtained by owners
Strength	Stair climb	M&M	Continuous numeric value (seconds) obtained by owners
Cognitive performance	Working memory	Cognitive task	Continuous numeric value (number of accurate trials) obtained by owners
	Learned behavior	CSLB	Ordinal qualitative description reported by owners
Mood and social relations	Anxiety/nervousness	HLES (C-BARQ)	Ordinal numeric score reported by owners
	Social avoidance	CSLB	Ordinal qualitative description reported by owners

### 3.1. Physical condition

Three different metrics of physical condition, body condition score (BCS), thigh girth deterioration, and unintentional weight loss, will be included in the initial DAP FIDo. Body condition score, a commonly used 1–9 scale used for the evaluation of an animal’s body fat from emaciated to obese, will be extracted from VEMRs. Increased BCS has been shown to be predictive of disease and mortality risk in canines (106), but a ‘protective effect’ associated with increased BCS has also been reported in the study of a canine frailty index model (45). Sarcopenia is an increasingly important factor in the assessment of human frailty and the mechanism by which it occurs is receiving increasing scrutiny (19, 107–109). As a component of frailty or an isolated finding, sarcopenia has been shown to be a marker for increased risk for disease and death (109). Sarcopenia has been described in the dog (110, 111) and a clinical assessment, the Muscle Condition Score (MCS) (111, 112), is available in veterinary practice. However, MCS is not yet a widely utilized tool in general practice and is found in only 5% of VEMRs submitted to the DAP. By contrast, it has been shown that minimally trained owners can obtain measurements of their dogs’ thigh circumference with strong agreement to those obtained by veterinary researchers (113). Thus, the DAP FIDo will use serial thigh circumference measurements to assess canine sarcopenia. Unintentional weight loss is a consistent factor in human frailty instruments. DAP participating owners provide their dogs’ weights annually (once in HLES, and subsequently in AFUS). We will also extract weights recorded at veterinary visits from VEMRs. As is the case for people, intentional weight loss in adult dogs is difficult for most owners to achieve (114, 115) the assumption will be that any detected weight loss was unintentional. However, the variable intervals at which weights are recorded at veterinary visits, and the difficulty of confirming whether any detected weight loss was truly unintentional, may preclude the value of this factor; validation analysis will determine whether it remains in the final instrument.

### 3.2. Physical activity

A variety of metrics of physical activity including objective kilocalorie expenditure, as well as self-reported activity, inactivity, exhaustion, and fatigue, are heavily utilized in human frailty instruments due to their predictive value. Importantly, these metrics are designed to detect or assess the individual’s customary amount and vigor of activity (15, 56, 86, 116), rather than the peak performance of which an individual is capable. Companion dogs often depend upon their owners for physical activity, such as being taken on leashed walks, or being given access to a park for play. For this reason, a simple assessment of the frequency of activity may reflect owner preferences more than dog ability. By contrast the dog’s interest in activity, vigor of activity, and change in activity patterns over time may be better suited to detect the onset of frailty. These topics of dog activity are addressed in HLES and AFUS, and we will derive a measurement of physical activity from owner responses to questions including, “Please choose the best description of your dog’s lifestyle over the past year. [not active/moderately active/very active],” “Over the past year, on average how much time per day is your dog physically active? Number of hours active: [0–8+], Number of minutes active: [0/10/20/30/40/50],” and “Over the past year, when your dog is active, what is the average intensity level of that activity? [low(walking)]/moderate [jogging]/[vigorous (sprinting)].” Other follow-up items identify the nature of the activity, including environmental conditions, on vs. off-leash, type of activity, etc. The use of multiple questions surrounding owner-reported activity allows for us to find the components that most accurately reflect the change in a dog’s activity as well as its relation to frailty.

### 3.3. Mobility

Despite significant variability between instruments used in human medicine, there is an overarching consensus that a measure of physical function is needed (117–119). A common measurement of mobility in human frailty instruments is walking speed, where an individual’s time to walk, a specified distance is compared to the lowest 20% of the population (15). Slower walking speeds in people have been shown to be predictive of worse postoperative outcomes (120–122), morbidity, and mortality (122–124); similar results were seen in rodents (125–127). Several small studies of mobility have shown a general trend of decline in functional capacity and spontaneous activity with but results also varied by location, breed and sex (128–130). Mobility scales for use in companion dog populations have previously been proposed, but they have not been validated or implemented in large populations (131, 132).

When developing a canine mobility scale Gonçalves et al. found a statistically significant difference in mobility scale scores between age quartiles (131), however, the presence of orthopedic and neurologic diseases was also noted to produce a statistically significant difference in scores (131). Banzato et al.’s frailty index study also reported poor mobility and low physical activity were significantly associated with time to death independently (45).

Morgan et al. morphologic and mobility trials found age was a weak but significant predictor of a dog’s speed for a given height (113), suggesting speed could be a useful frailty factor. They also showed that owners were able to perform low-tech assessments of their dogs’ speed with minimal training and were able to obtain measurements that strongly agreed with those obtained by investigators (113). Consequently, the DAP FIDo will include times to traverse a measured distance on-leash and off-leash over a flat-surface from our Measurement and Mobility instrument. A greater magnitude of change in off-leash speed with increasing age was reported (109), but this metric may not be a significant indicator of frailty among dogs of the same age. Thus, both on- and off-leash flat-surface speed will be evaluated separately in the initial model to determine whether either or both add predictive value to the frailty model independently of age alone.

### 3.4. Strength

Strength is also frequently included in human frailty instruments. In humans, strength can be assessed utilizing a variety of measurements including the ability to rise from a chair (39) and stair climbing (133); grip strength (15, 87) is the most popular measure. Grip strength is not a feasible metric in dogs, but stair climbing is a routine and accessible activity for many dogs. Elderly people often become reluctant to climb stairs as they age (127–129) and the authors’ clinical experience suggests that the same is true among companion dogs. Morgan et al.’s morphologic and mobility trials also assessed timed stair ascent and found that age was a weak but significant predictor of speed in this task as well (113). Other options that were considered as strength assessments, such as pressure-sensing chew toys (134), or systems that measure the strength with which a dog can pull against a measurement device (135, 136), are expensive, have limited availability, depend upon the dog’s training and interest, or some combination of those flaws. The DAP FIDo will use timed stair ascent as our measurement of canine strength.

### 3.5. Cognitive task performance

Cognitive decline is an important factor in many human frailty instruments (33, 137–139). There is an incompletely understood relationship among frailty, mild cognitive impairment, and the ability to perform the Instrumental Activities of Daily Living (IADL) in humans (55, 92, 140–142). While IADLs cannot readily be extrapolated to dogs, simple cognitive tasks that mimic daily activities have been developed and validated in dogs. Dogs, like humans, display evidence of mild cognitive decline with age, and their performance on these tasks also declines with age (143, 144). We will use performance on a purpose-built at-home cognitive task designed to assess working memory within our DAP FIDo. The purpose-built cognitive task is easily administered at home and does not require specialized equipment or dog training. Dogs are tested for their ability to (1) recall the location of a food item after varying delays [delayed search], or to recall which location still contains a food item after consuming food items from all other locations [radial array]. Instructions are provided through online video tutorials and owners respond to simple questions about their dog’s behavior through an interactive online survey.

Like humans, some dogs also develop dementia, called Canine Cognitive Dysfunction Syndrome (CCDS) (145, 146). The Canine Cognitive Dysfunction Rating Scale (CCDRS) is a validated survey used to diagnose CCDS among dogs with a cumulative score of 50 or greater (104, 105, 147). We have deployed this instrument within our DAP population as an annual survey, rebranded the Canine Social and Learned Behavior (CSLB) instrument, to avoid potential negative connotations of the diagnostic term, “cognitive dysfunction.” However, the purpose of a frailty instrument is to detect early, mild change in function, before an overt diagnosis, such as CCDS, is made. Thus, we will use individual items from the CSLB, rather than the total score, within our DAP FIDo. Specifically, we will use responses to the items “How often does your dog stare blankly at the walls or floor?” and “Compared with 6 months ago, does your dog have difficulty finding food dropped on the floor?” to detect changes in at-home cognitive performance over time. The total CSLB score, as well as owner-reported diagnoses in the Health Section of HLES, will be evaluated to ensure that the selected CSLB items do not reflect a diagnosis of CCD or another neurologic disease that may have its own impact on mortality.

### 3.6. Social behavior

Recent work in human frailty has promoted the importance of including social components in frailty assessments (1, 148). While questions about happiness and social support networks cannot be readily extrapolated to dogs, there is a large body of research describing normal companion dog behavior, including socially interactive behavior (149–152). The Canine Behavioral Assessment and Research Questionnaire (C-BARQ) is a validated survey used to objectively document companion dog behavior in a variety of settings (153–155). We have deployed the C-BARQ as the Behavior component of HLES and AFUS, so that it is completed annually by DAP participants. We will use responses to specific items from both C-BARQ and CSLB to represent mood and social relations in the DAP FIDo. Specifically, we will use the cumulative score from the Fear and Anxiety section of C-BARQ and the item on social avoidance, “How often does your dog walk away while, or avoid, being petted?” from CSLB.

These items addressing physical condition, physical activity, mobility, strength, cognitive task performance, and mood and social relations will be included in the first version of the DAP FIDo to identify those domains and specific factors that most strongly predict mortality among companion dogs. The model will be revised to include the fewest, but most informative elements, to facilitate wide deployment among companion dog owners and veterinary practices.

## 4. Discussion and conclusion

The assessment of frailty plays a central role in human gerontology as a superior means to understand, describe, and mitigate certain manifestations of aging and the associated risks of poor health outcomes, including death within individuals and communities. The numerous frailty instruments for humans that have been developed include diverse domains and factors within each domain, leading to challenges in comparing the efficacy and utility of these instruments across populations and settings. As companion dogs increasingly survive into geriatric ages, the ability to document their frailty, and to understand its impact on health outcomes, becomes increasingly valuable. The Dog Aging Project is collecting targeted data to enable the construction and validation of a companion dog frailty instrument designed to be uncomplicated, quick to complete, low-tech, low-cost, and accessible to dog owners and veterinary general practitioners. Use of this tool, once finalized, will enhance both research opportunities, and the ability to provide excellent veterinary care, to aging companion dogs.

## Author contributions

AF, AR, KC, NO, and RM worked together to construct an outline of the manuscript. RM wrote the first draft of the manuscript. AR, NO, AF, and KC revised and edited sequential drafts of the manuscript. EP contributed to the visualization and revision. All authors have read and approved the final manuscript.

## Funding

The Dog Aging Project is supported by U19 grant AG057377 from the National Institute on Aging, a part of the National Institutes of Health, and private donations. The content is solely the responsibility of the authors and does not necessarily represent the official views of the National Institutes of Health.

## Conflict of interest

The authors declare that the research was conducted in the absence of any commercial or financial relationships that could be construed as a potential conflict of interest.

The reviewer BM declared a past co-authorship with the author NO to the handling editor.

## Publisher’s note

All claims expressed in this article are solely those of the authors and do not necessarily represent those of their affiliated organizations, or those of the publisher, the editors and the reviewers. Any product that may be evaluated in this article, or claim that may be made by its manufacturer, is not guaranteed or endorsed by the publisher.
